# Ruptured renal artery aneurysm during pregnancy, a clinical dilemma

**DOI:** 10.1186/1471-2490-6-22

**Published:** 2006-08-31

**Authors:** Khaled B Soliman, Yaser Shawky, Mohamed M Abbas, Mohamed Ammary, Allaa Shaaban

**Affiliations:** 1 Obstetrics Gynecology Department, Maternity and Children Hospital, Buraidah, Qassim, Saudi Arabia; 2 General Surgery Department, King Fahd Specialist Hospital, Buraidah, Qassim, Saudi Arabia; 3 Urology Department, King Fahd Specialist Hospital, Buraidah, Qassim, Saudi Arabia

## Abstract

**Background:**

Rupture of a renal artery aneurysm (RAA) during pregnancy is a rare event, with a high mortality rate for both mother and fetus. Increased blood flow and intra-abdominal pressure, and vascular changes secondary to increased steroid production are postulated as contributory to the increased risk of rupture during pregnancy.

**Case presentation:**

We present here a case report of total avulsion of solitary kidney secondary to rupture of RAA in a pregnant patient with congenital absence of the contralateral kidney. The main indication for nephrectomy was severely damaged kidney. Diagnosis was made during operation and both mother and fetus were saved. There are no previous reports of an intact renal artery aneurysm diagnosed either antepartum or postpartum.

**Conclusion:**

The possibility of a ruptured RAA should be considered in pregnant women with evidence of retroperitoneal hemorrhage. This case was unusual because it occurred in a solitary kidney, during the third trimester of pregnancy.

## Background

Renal artery aneurysm (RAA) occurs due to focal dilatation of the artery secondary to weakness of the arterial intima and media. In most of the cases it is present as a small (1–2 cm) and unilateral one. Renal artery aneurysm is a rare clinical entity with an incidence of 0.015–1%. The aetiology of RAA is either, congenital or acquired secondary to atherosclerosis, malignancy, etc. Solitary kidney is an abnormally heavy one, due to compensatory hypertrophy and also it is more liable to have other congenital anomalies like RAA. Rupture or total avulsion of the kidney secondary to rupture RAA during pregnancy is an extremely rare event, even more the obstetric and urological literatures are Deficient in this regard. Predisposing factors of rupture RAA are, incomplete calcification, size > 2 cm, and presence of hypertension and if pregnancy co-exists. Diagnosis of rupture RAA during pregnancy is very difficult as there is no path gnomonic pain or presentation. When rupture occurs during pregnancy the clinical presentation is easy to be confused with those more common conditions like placental abruption or rupture uterus. Most of the cases are discovered incidentally; even sometimes it is only diagnosed after autopsy. When rupture RAA occurs during pregnancy it carries a high fatality or poor feto-maternal outcomes. When RAA is diagnosed during pregnancy the treatment modalities, are conservative if non calcified and small < 2 cm or aneurismal resection and vascular reconstruction, end to end anastomosis or nephrectomy if the patients has extensive renal injury or haemodynamically unstable or it is usually done as a life saving procedure.

## Case presentation

A 40 year old woman, gravida 10 para 9, unbooked, presented to the emergency room, severely ill, complaining of severe acute abdominal pain since 1 hour. According to her given expected date of confinement, she was 35 weeks and four days of gestation. On admission, she had no history of medical disorders or trauma. Generally, she was very pale, conscious, agitated, hypotensive and with severe tachycardia (Pulse 120/bpm; BP 90/50 mmHg, Temp 37.5°C). Abdominally, marked tenderness and rigidity all over the abdomen was found, fetal heart sounds showed severe bradycardia 100/bpm. Vaginal examination revealed no bleeding and the cervix was closed, firm and posterior. Laboratory studies, included a complete blood cell count, chemistry, coagulation profile, and arterial blood gases, were undertaken. In view of her clinical presentation, as BP was decreasing and the pulse was increasing, the provisional diagnosis was query placental abruption or rupture uterus. Rapid infusion of 2 L of crystalloid was started. Cesarean section was done immediately, there was no signs of placental abruption as the placenta was upper, posterior and fully attached to the uterine wall. She gave birth of a baby boy (2.2 Kg), Apgar score (1,6,8), admitted to the NICO. After closure of the uterus, the pelvis and abdomen were explored. A huge retroperitoneal haematoma was found extending behind the descending colon. The general and vascular surgeons were called, they advised conservative treatment as the haematoma was neither expanding nor pulsating. The abdomen was closed and 2 intra-peritoneal drains were left. During the operation she received 4 units FFP, 2 units blood 4 units of packed cells and remained haemodynamically stable. The patient was kept in recovery room for close monitoring. Within one hour BP dropped again to 100/60 mm Hg, pulse increased to 110/bpm, blood was coming from the drains and the patient was deteriorating. Both surgeons were called again for re-assessment; they decided to re-explore the abdomen. The retroperitoneal haematoma was found to be expanded via the left paracolic gutter, the posterior peritoneum over the haematoma was opened. A voluminous retroperitoneal haematoma (about 3000 ml blood and clot), due to an undiagnosed totally avulsed left kidney, was found. There was severe bleeding from the hilar vessels. The bleeding vessels were clamped and the urologist was called and shared the operating team. A decision of nephrectomy was taken, after a committee, as the left kidney was severely damaged and as a life saving procedure. The urologist tried to feel the other kidney but he failed because of the extensive haematoma. During re-exploration she received, 6 units packed cells, 6 units FFP and IV fluids. After closure of the abdomen the patient was found to be anuric. The urologist preferred to do cystoscopic examination versus right ureteric catheterization but he failed to visualize the right ureteric orifice. Abdominal and pelvic ultrasound was done at the theatre and failed to visualize the right kidney. The patient was transferred to ICU, anuric, under sedation on ventilator. She stayed anuric for 2 days in the ICU, after that she was transferred to the postoperative ward. Her postpartum recovery was uneventful apart from progressively increasing kidney function tests. CT was done in the second postoperative day and confirmed the absence of right kidney. She was discharged after 10 days from admission and a haemodialysis twice sitting per week was scheduled for her. The patient now is waiting for compatible kidney transplant. Histopathological report revealed degenerative aneurysmal changes of the renal artery and acute tubular necrosis of the left kidney.

## Discussion

We reported on a pregnant patient at 35 weeks of gestation, who had total avulsion of a solitary kidney secondary to spontaneous rupture of RAA, treated by nephrectomy with favorable outcomes.

To our knowledge this is the first case report in the literature, of total avulsion of a solitary kidney late in pregnancy.

The natural history of the RAA, is not known with certainty, but it has potential complications including embolization and rupture [1]. Renal artery aneurysm is a rare condition, with an incidence ranging between 0.01% and 0.09%, and accounts for 1% of all aneurysms [2,3]. Risk factors for rupture include incomplete calcification, size >2 cm, progressive enlargement and pregnancy. Rupture of RAA in a kidney during pregnancy is a rare and well described catastrophic event, with a high mortality rate for both mother and fetus [2,4]. Renal artery aneurysm is diagnosed more frequently in recent years mainly due to the extensive use of angiography. Reconstruction and repair of these aneurysms, with preservation of the kidney, is the preferred treatment [5].

Prior to 1970, reported rupture of RAA occurred most often on the left side, during the third trimester, and was associated with a 92% maternal mortality and a 100% fetal mortality rate. Since 1970, published reports have not shown a left-sided predominance, and survival after this catastrophic event has greatly improved, even though preoperative diagnosis is still rare [6].

Twenty six cases of RAA during pregnancy have been reported in the English medical literature. Renal salvage with in situ repair of the renal artery has been documented in only four cases, and successful ex situ repair and auto transplantation in only two cases [4,7,8].

There are no previous reports of diagnosis of an intact RAA during pregnancy and only one postpartum case was diagnosed and treated successfully by Pliskin et al. [9]. They reported a giant right RAA diagnosed intact, 8 weeks postpartum which was repaired successfully with kidney salvage and closure of the fistulous connection to the renal vein.

Martin et al. [10], reported thirty-nine patients with RAA, which were seen over a period of 15 years. Among the 20 women of their series, nephrectomy was performed in two patients with ruptured RAA at the time of childbirth and in one patient with hypertension and RAA in a poorly functioning kidney.

The clinical presentation of our case was easily confused with those more common conditions, as she was thought to have either a placental abruption or ruptured uterus. Satoh et al. [11], reported a case of spontaneous rupture of the renal pelvis during pregnancy, at 20 weeks of gestation. The patient was initially mis-diagnosed, like our case, as a case of renal/ureteral calculi. She had no further urological problems during pregnancy and a male infant was delivered at 41 weeks' gestation.

During re-exploration the urologist failed to palpate the contralateral kidney from the extensive retroperitoneal haematoma she had. Dayton et al. [8], reported that in case of ruptured RAA during pregnancy, the retroperitoneal anatomy may be severely distorted by haematoma. It may be nearly impossible to determine the exact anatomy of renal vessels and the presence or absence of the contralateral kidney at the time of surgery.

Spontaneous rupture of RAA is more likely to occur during pregnancy and when it does, there is an associated high mortality for the mother and fetus. Increased blood flow and intra-abdominal pressure, and vascular changes secondary to increased steroid production are postulated as contributory to the increased risk of rupture of RAA during pregnancy [12].

In a trial to solve the puzzle, what are the causes of total avulsion of the kidney in the current pregnancy? We proposed the following: (1) Our patient was grand multipara, and it is well known that, every single pregnancy does anatomical and physiological changes in the kidney. (2) The physiological changes gave the impression of obstructive uropathy. (3) Solitary kidney, is an abnormally heavy one, due to compensatory hypertrophy and also it is more liable to have other congenital anomalies like RAA. (4) The effect of hyperdynamic circulation on an already existing RAA together with an abnormal solitary kidney. (5) The resultant increase intra-ureteral pressure acts as an axis, in addition, to the presence of an abnormal heavy kidney (*Dancing effect*), make the kidney more liable to injury during pregnancy. Franz et al. [13] concluded that abnormal kidneys are more vulnerable, to be injured than normal kidneys.

Surgical treatment of an intact RAA, prevents the occurrence of ruptures as well and gives long term satisfactory anatomical results. Surgery, preferably primary repair is indicated in most cases and especially in young women (because of the risk of rupture during pregnancy) and in aneurysms exceeding 2 cm in diameter [12,14].

Lumsden et al. [1], concluded that the indications for surgery are: (a) symptomatic or enlarging aneurysms; (b) renal embolization; (c) aneurysms in pregnant females or those considering pregnancy; (d) renovascular hypertension; (e) aneurysms > 2.5 cm. Other patients with asymptomatic RAA can be safely observed clinically without serial arteriograms and without fear of rupture [8].

## Conclusion

The possibility of a ruptured RAA and/or kidney, should be considered in pregnant women with evidence of retroperitoneal hemorrhage. This case was unique because, the spontaneous rupture of RAA resulted in total avulsion of the solitary kidney.

## Competing interests

The author(s) declare that they have no competing interests.

## Authors' contributions

Khaled B. Soliman. MT, FG Yaser Shawky., FG Mohamed M. Abbas., FG Mohamed Ammary. FG, Allaa Shaaban. FG. All authors read and approved the final manuscript.

## Pre-publication history

The pre-publication history for this paper can be accessed here:


